# Upregulation of BRCA1 and 2 protein expression is associated with dysregulation in amino acids profiles in breast cancer

**DOI:** 10.1007/s11033-023-09028-6

**Published:** 2024-01-02

**Authors:** Tahia H. Saleem, Mohamed A. Rizk, Nashwa F. Abdelhafez, Ahmed Sabra, Eman Radwan

**Affiliations:** 1 Medical Biochemistry Department, Faculty of Medicine, Assiut University, Assiut, Egypt; 2 General Surgery Department, Faculty of Medicine, Assiut University, Assiut, Egypt; 3 Anesthesia and Intensive Care Unit, Faculty of Medicine, Assiut University, Assiut, Egypt; 4 Medical Biochemistry Department, Faculty of Medicine, Merit University, Sohag, Egypt; 5 Biochemistry Department, Sphinx University, New Assiut, Assiut, Egypt

**Keywords:** Breast cancer, BRCA, Amino acids

## Abstract

**Background:**

The prevalence of breast cancer (BC) is high among cancers in Egypt, ranking it the most common cause of cancer mortality in women. BRCA1 and BRCA2 tumor suppressors proteins have a specific relationship with BC. Plasma free amino acids levels (PFAAs) have been reported to exhibit altered profiles among cancer patients. Thus, the present study aims to examine the alteration of the PFAAs profiles and investigate their association with BRCA1 and 2 circulating levels in Egyptian females diagnosed with BC and in females with family history of BC to establish potential early detection strategies for BC.

**Methods and results:**

This study included 26 BC patients, 22 females with family history of BC (relatives) in addition to 38 healthy females as control group. Quantitative measurement of PFAAs was determined by the ion exchange separation method through high performance liquid chromatography. BRCA1 and BRCA2 concentrations were determined using ELISA. Our results showed PFAAs profiles in BC patients and in females with BC family history with significant upregulation in mean plasma levels of Alanine, Phenylalanine, Glutamate and Cysteine and downregulation of Taurine, Threonine, Serine, Glycine, Valine, Methionine and Histidine levels compared to controls. Also, a significant positive correlation was observed between plasma BRCA1 and Valine levels while a significant negative correlation was observed between BRCA2 and Lysine plasma levels.

**Conclusion:**

PFAAs profile can potentially be used in early screening for BC patients and for susceptible females.

## Introduction

Breast cancer (BC) is the most common malignancy in females. In 2020, more than 2 million new cases were diagnosed worldwide [1, 2]. The prevalence of breast cancer (BC) is high among cancers in Egypt, ranking it the most common cause of cancer mortality in women [3, 4].

Several risk factors for BC are identified. Advanced age, female sex and family history are among the most common factors [5]. In addition, mutations of BRCA 1 and 2 genes account for a significant number of BC cases [6].

BRCA1 and BRCA2 proteins are tumor suppressors that play an important role in DNA damage response and repair. BRCA1 is also needed for estrogen receptor (ER) gene transcription [7]. Upregulated expression of BRCA1 and BRCA2 genes was reported in BC and ovarian cancer, furthermore, high BRCA1 gene expression was demonstrated to increase the risk of early distant metastasis in ER + breast cancer patients [8, 9].

Although BC survival rates have significantly improved recently, however, they remain considerably low in Egypt. This may be attributed to deficient screening mechanisms among Egyptian females where most patients are reported to being diagnosed at late stages with subsequent poor outcomes [10–13].

Several efforts have been made recently to develop low cost, rapid, easy-to-use cancer diagnostic methods with minimal invasiveness using peripheral blood or urine samples [14]. Metabolome analysis has emerged as an effective tool in cancer diagnosis and prognosis [14, 15].

Amino acids (AA) are an integral part of most metabolomics analysis [16]. Amino acid profiling has demonstrated significant differences in AA levels between cancer patients and healthy controls [14]. Several studies reported a decrease in AA plasma levels in cancer cells probably attributed to their high requirements in cancer cells [17, 18]. However, other studies showed an increase of amino acids levels in tumors which could be a result of the increased cellular proliferation rate [17, 19].

To date, amino acid profiling of Egyptian women with BC hasn’t been well investigated, so we aimed in the present study to examine the alteration of the plasma amino acids profiles and investigate their association with BRCA1 and 2 circulating levels in Egyptian females diagnosed with BC and in females with family history of BC in order to establish potential novel non-invasive screening strategies which will help in the early detection of BC and provide insight into the progression of the disease using a safe, low cost and easy method.

## Materials and methods

### Patients

The present study is a case control study conducted in the departments of Biochemistry, Faculty of Medicine, Assiut university and General Surgery, Assiut University Hospital, Assiut, during the period from March 2021 to December 2022.

The sample size was calculated using the Steven K. Thompson equation (Thompson, S.K., 1987) to be a total size of 86 samples. We adjusted the sample size to attain 80% power and 5% confidence level of significance (type 1 error)

$$N = \frac{{N \times P\left( {1 - P} \right)}}{{\left[ {N - 1 \times \left( {{d^2} \div {z^2}} \right) + P\left( {1 - P} \right)} \right]}}$$

*N =* population size, d = margin of error, *p* =  *p* value; 0.05 and z = confidence level at 96% is 1.96.

The study included 86 participants that were subdivided into three groups as follows:

#### Group I

consisted of 26 females who were diagnosed with BC.

#### Group II

consisted of 22 females who have a family history of BC (relatives of group I).

#### Group III

consisted of 38 healthy females (control).

An informed consent was obtained from each subject and all study procedures were approved by the Medical Ethics Committee, Faculty of Medicine, Assiut University (IRB no: 04-2023-200140). The aim of the study was explained to each participant before filling data. Personal data including age, weight, height, and BMI were collected during time of admission. Medical history including duration of symptoms, medication used, and presence of other complications associated with symptoms were also collected from patients’ profiles.

Exclusion criteria included females diagnosed with any other type of cancer (benign or malignant), females that past received a chemotherapy or radiotherapy, females with hypertension or diabetes and females who have a history of coronary artery disease, stroke or myocardial infarction.

### Sample collection and handling

Samples were collected from patients, relatives and controls. Samples of five milliliters of antecubital venous blood were collected and divided into: Three milliliters of blood which were placed in a tube containing heparin for plasma separation for AAs profile assessment. Another two milliliters were placed in a plain test tube and centrifuged at 3000 rpm for 10 min, then serum was separated and stored at -80˚C until time of ELISA analysis.

### Estimation of BRCA1 and BRCA2

BRCA1 and BRCA2 concentrations were determined using commercial ELISA Kits (Catalog Numbers: SG-14,409 and SG-15,000 respectively) purchased from SinoGeneclon Biotech Comp, Hangzhou, China according to the manufacturer’s specifications.

### Assay of amino acids profiles

**Fig. 1:**
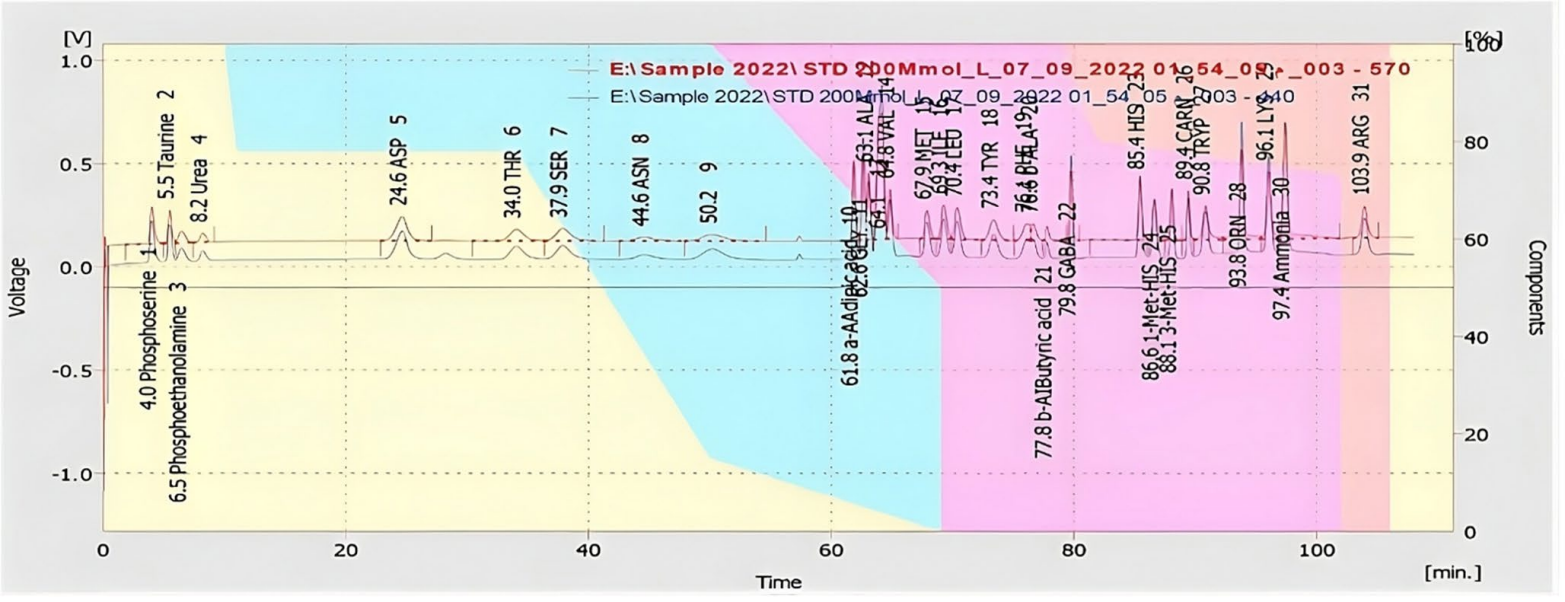
Chromatogram of standard amino acids showing their retention times (minutes) and voltage (volt) response peaks. MIN, minute; V, volt; ASP, Aspartate; THR, Threonine; SER, Serine; ASN, Aspargine; GABA, Gamma Amino Butyric Acid; GLY, Glycine; ALA, Alanine; VAL, Valine; MET, Methionine; ILE, Isoleucine; LEU, Leucine; TYR, Tyrosine; PHE, Phenyl Alanine; HIS, Histidine; CARN, Carnitine; TRYP, Tryptophan; ORN, Ornithine; LYS, Lysine; ARG, Arginine

Assay of amino acids was performed in the Metabolic and Genetic Disorder Unit (MGD) of Faculty of Medicine, Assiut University by the Ion exchange separation method through high performance liquid chromatography using a Sykam Automatic Amino Acid Analyzer S433 supplied by Sykam GmbH, Germany (catalog no. 1,120,001). By using an acidic protein precipitation method, free amino acid samples were made from plasma by adding 200 µl of a 10% sulfosalicylic acid solution to 800 µl of plasma, mixing by vortex, and allowing it cool to about 4 °C for 30 min. It was then centrifuged at 14,000 rpm for 10 min. The same amount of sample dilution buffer (catalog no. S000015) was added to the supernatant liquor to dilute it. Direct injections of 100 µl of each of prepared samples and ready to use amino acid physiological standard (Catalog no. 6,006,005) were made. A cation separation column LCAK07/Li was used (catalog no. 5,112,008) with the following specifications: size: 150 mm × 4.6 mm, specification range: methionine (Met) efficiency > 48,000, asymmetry: 0.8–1.5, resolution THR/SER: > 1700, and column pressure: 45–80 bar. Buffer: Sykam Li A, Li B, Li C. The ready to use ninhydrin reagent (catalog no. 8,000,025) and citrate buffers in different pH (2.90, 4.20, and 8.0) were used and we performed the analysis at wavelength 440 nm:570 nm. The sample chromatogram was then compared to the standard measurements curve to obtain various amino acid values, then results were multiplied by a dilution factor of 2.5 (Fig. 1).

### Statistical analysis

Data entry and analysis were done using SPSS version 22 (Statistical Package for Social Science). Data were presented as mean, standard deviation or median and range. In case of parametric data, one way ANOVA test followed by post-hoc test was used to compare between different groups. While in case of non-parametric data; Kruskal Wallis test was used to compare between different groups and spearman correlation was done to measure correlation between quantitative variables. P-value considered statistically significant when less than 0.05.

## Results

### Personal data of subjects

The personal data of the three groups of subjects are shown in Table 1. There was no statistically significant difference regarding the age, height, weight, or BMI between the study groups.

**Table 1:** Personal data of different study groups

Personal data	Group I (*n* = 26)	Group II (*n* = 22)	Group III (*n* = 38)	*P*- value^1^	*P*- value^2^	*P*- value^3^	*P*- value^4^
**Age:**							
Mean ± SD	44.92 ± 5.49	43.27 ± 4.22	44.37 ± 9.02	0.717	0.422	0.758	0.564
Range	30.0–53.0	36.0–53.0	25.0–60.0				
**Weight**:							
Mean ± SD	64.58 ± 8.95	65.41 ± 9.17	64.11 ± 4.88	0.810	0.702	0.805	0.517
Range	50.0–87.0	50.0–88.0	52.0–75.0				
**Height**:							
Mean ± SD	159.04 ± 5.50	160.73 ± 7.75	160.84 ± 6.24	0.511	0.369	0.276	0.947
Range	149.0-167.0	145.0-171.0	149.0-173.0				
**BMI**:							
Mean ± SD	25.47 ± 2.77	25.30 ± 2.87	24.83 ± 2.11	0.580	0.812	0.323	0.494
Range	20.6–32.0	21.7–32.3	21.0-29.3				

**P1**: Comparison between all groups. **P2**: Comparison between group I and group II. **P3**: Comparison between group I and group III. **P4**: Comparison between group II and group III. One way ANOVA test followed by post-hoc test/LSD was used to compare between different groups

### Clinical data of BC patients

Table 2 shows the clinical data of BC patients. The present study included 26 patients diagnosed with BC. 18 of the patients had BC of stage 2 and 8 of them had a stage 3 BC according to TNM staging. 84.5%. of the patients were at grade 2 of the disease and 80.8% did not have any family history of BC. Receptor status percentage differentiated between patients, where luminal A, luminal B and non-luminal cases were 42.3%, 38.5% and 19.2% respectively.

**Table 2:** Clinical data of BC patients

Variable	Number (Percentage)
**TNM stage**	
**Grade**	
Grade 1 (well differentiated)	*n* = 22 (84.6%)
Grade 2 (moderately differentiated)	*n* = 3 (11.5%)
Grade 3 (poorly differentiated)	*n* = 1 (3.85%)
**Family history**	
Positive	*n* = 5 (19.2%)
Negative	*n* = 21(80.8%)
**Hormone receptor status**	
Luminal (A)	*n* = 11(42.3%)
Luminal (B)	*n* = 10 (38.55%)
Non-luminal	*n* = 5 (19.2%)
Age of presentation	(50–53) years: *n* = 9 (34.6%)
	(40–50) years: *n* = 11(42.3%)
	(30–40) years: *n* = 5 (19.2%)
	28 years: *n* = 1(3.84%)

### BRCA1 and BRCA 2 levels

Table 3 shows the mean levels of BRCA1 and 2 in serum where a significant difference in BRCA1 levels was detected between both (groups 1& II) compared to group III. No statistically significant difference was found in mean BRCA1 level between the patients and their relatives. In addition, a significant difference was found between the mean BRCA2 levels in the three groups as it was significantly higher in patients compared to the relatives’ and control groups. Also, there was a significant increase in mean BRCA2 levels in relatives compared to controls.

**Table 3:** BRCA1 and 2 concentrations in serum of different study groups

	Group I (*n* = 26)	Group II (*n* = 22)	Group III (*n* = 38)	*P*- value^1^	*P*- value^2^	*P*- value^3^	*P*- value^4^
**BRCA 1:**							
Mean ± SD	1638.16 ± 810.29	1660.61 ± 695.49	1275.62 ± 563.45	0.023*	0.885	0.018*	0.023*
Median (Range)	1824.7 (153.9-2654.3)	1736.7 (567.2-2910.5)	1425.1 (228.6-2352.4)				
**BRCA 2**:							
Mean ± SD	15.88 ± 4.13	14.27 ± 3.51	11.88 ± 3.87	0.000*	0.015*	0.000*	0.016*
Median (Range)	16.6 (5.1–21.6)	15.4 (4.1–17.3)	12.6 (5.4–17.3)				

**P1**: Comparison between all groups. **P2**: Comparison between group I and group II. **P3**: Comparison between group I and group III. **P4**: Comparison between group II and group III. Kruskal Wallis test followed by Mann-Whitney test were used to compare between different groups

### Plasma concentrations of amino acids in different study groups

There was a significant statistical difference in mean plasma levels of Phosphoserine, Alanine, Phenylalanine, Glutamate and Cysteine where their plasma levels in patients and relatives were higher compared to levels in the control group. We also noted that the levels of Glutamate were significantly higher in patients compared to females with family history of BC (Table 4). On the other hand, there was a significant statistical decrease in mean plasma levels of Taurine, Threonine, Serine, Glycine, Valine, Methionine and Histidine where their plasma levels were lower in patients and relatives compared to controls (Table 5). There was no statistical significance in mean plasma levels of Aspargine, Aspartate, Leucine, Isoleucine, Tyrosine, Ornithine, Lysine and Arginine between the three groups (Table 6).

**Table 4:** Amino acids that increased in BC patients compared to controls

	Group I (*n* = 26)	Group II (*n* = 22)	Group III (*n* = 38)	*P*- value^1^	*P*- value^2^	*P*- value^3^	*P*- value^4^
**Phosphoserine:**							
Mean ± SD	18.91 ± 3.96	16.66 ± 4.23	8.88 ± 1.48	0.001*	0.357	0.000*	0.009*
Range	11.7–24.8	12.0-20.2	7.4–10.7				
**Alanine (Ala)**:							
Mean ± SD Range	535.77 ± 91.52 448.3-660.8	472.41 ± 77.54 413.0-585.3	259.23 ± 51.14 176.2-427.8	0.000*	0.134	0.000*	0.000*
**Phenylalanine (Phe)**:							
Mean ± SD	79.26 ± 15.34	84.05 ± 18.26	58.63 ± 8.82	0.000*	0.522	0.000*	0.000*
Range	55.7–99.8	61.9-106.1	44.3–77.7				
**Cysteine (Cys)**:							
Mean ± SD	46.50 ± 11.37	45.56 ± 1.21	24.51 ± 15.90	0.011*	0.439	0.009*	0.043*
Median (Range)	52.0 (30.8–57.9)	45.3 (44.5–46.9)	25.1 (6.6–52.1)				
**Glutamate** **(Glu)**:							
Mean ± SD	491.58 ± 72.61	317.62 ± 136.21	67.99 ± 14.00	0.000*	0.038*	0.000*	0.002*
Median (Range)	489.1 (402.6-601.8)	348.7 (135.1–438.0)	67.3 (51.6–96.8)				

**P1**: Comparison between all groups. **P2**: Comparison between group I and group II. **P3**: Comparison between group I and group III. **P4**: Comparison between group II and group III. One way ANOVA test followed by post-hoc test/LSD were or Kruskal Wallis test followed by Mann-Whitney test were used to compare between different groups

**Table 5:** Amino acids that decreased in BC patients compared to controls

	Group I (*n* = 26)	Group II (*n* = 22)	Group III (*n* = 38)	*P*- value^1^	*P*- value^2^	*P*- value^3^	*P*- value^4^
**Taurine(Tau):**							
Mean ± SD	49.43 ± 10.16	45.27 ± 5.78	176.60 ± 131.72	0.024*	0.345	0.031*	0.044*
Median(Range)	50.9 (30.9–62.9)	43.5(40.9–53.1)	133.1 (38.0-382.8)				
**Threonine(Thr**							
Mean ± SD	125.60 ± 46.86	150.76 ± 49.99	162.39 ± 35.05	0.141	0.450	0.041*	0.757
Median(Range)	121.9 (61.2-208.4)	156.7(85.1-204.5)	156.7 (93.5-239.6)				
**Serine (Ser)**:							
Mean ± SD	105.83 ± 27.78	122.34 ± 45.91	167.47 ± 24.09	0.001*	0.450	0.000*	0.036*
Median(Range)	92.3 (78.0-146.5)	130.8(63.0-164.9)	167.5 (101.8-210.1)				
**Glycine (Gly)**:							
Mean ± SD	208.44 ± 57.44	220.48 ± 80.52	305.35 ± 50.25	0.003*	0.850	0.002*	0.044
Median(Range)	222.0 (138.4-280.9)	192.4 (160.6-336.6)	295.5 (233.6-394.1)				
**Valine (Val)**:							
Mean ± SD	155.94 ± 33.22	141.55 ± 32.74	229.96 ± 47.09	0.000*	0.597	0.001*	0.001*
Range	115.6-209.5	107.0-174.6	147.4-322.9				
**Methionine**							
Mean ± SD Median(Range)	60.91 ± 26.03 68.5 (22.3–95.4)	65.55 ± 32.66 70.1 (24.0-98.1)	80.99 ± 11.49 77.5 (69.1-108.4)	0.087	0.705	0.020*	0.588
**Histidine(His)**:							
Mean ± SD	68.59 ± 17.03	75.18 ± 13.48	85.11 ± 10.22	0.016*	0.405	0.006*	0.158
Range	48.9–98.8	62.5–93.7	62.5-101.2				

**P1**: Comparison between all groups. **P2**: Comparison between group I and group II. **P3**: Comparison between group I and group III. **P4**: Comparison between group II and group III. One way ANOVA test followed by post-hoc test/LSD were or Kruskal Wallis test followed by Mann-Whitney test were used to compare between different groups

**Table 6:** Amino acids that showed no difference in BC patients compared to controls

	Group I (*n* = 26)	Group II (*n* = 22)	Group III (*n* = 38)	*P*- value^1^	*P*- value^2^	*P*- value^3^	*P*- value^4^
**Aspargine(Asn):**							
Mean ± SD	93.15 ± 28.26	120.39 ± 41.50	81.41 ± 30.33	0.142	0.257	0.293	0.075
Median Range	93.6 (47.8-135.1)	126.4 (67.1-161.6)	73.6 (39.0-148.3)				
**Isoleucine (Ile)**:							
Mean ± SD	95.82 ± 31.73	93.67 ± 25.13	92.45 ± 11.94	0.925	0.862	0.696	0.909
Range	69.1-146.9	65.3-120.7	73.1-113.8				
**Leucine (Leu)**:							
Mean ± SD	154.84 ± 53.13	143.00 ± 36.01	153.93 ± 18.57	0.800	0.551	0.948	0.528
Range	100.3-237.3	101.5-181.4	117.3-184.7				
**Tyrosine (Tyr)**:							
Mean ± SD	76.23 ± 19.69	82.88 ± 27.71	84.94 ± 22.39	0.684	0.641	0.388	0.869
Range	47.5–105.0	59.5–123.0	48.3-130.4				
**Ornithine (Orn)**:							
Mean ± SD	64.18 ± 19.52	70.37 ± 20.44	118.95 ± 75.40	0.148	0.571	0.068	0.314
Median(Range)	64.2 (41.3–96.2)	72.2 (44.5–92.7)	81.8 (49.2-251.8)				
**Lysine (Lys)**:							
Mean ± SD	152.54 ± 41.73	180.44 ± 82.38	152.38 ± 26.32	0.779	0.571	0.580	0.699
Median(Range)	134.4 (117.2-228.5)	157.2 (108.4-298.9)	147.9 (107.9-201.7)				
**Arginine (Arg)**:							
Mean ± SD	101.89 ± 48.27	104.37 ± 27.24	111.24 ± 28.89	0.789	0.522	0.555	0.777
Median (Range)	104.3 (48.2-175.9)	108.9(71.2-128.4)	112.5 (64.2-176.4)				

**P1**: Comparison between all groups. **P2**: Comparison between group I and group II. **P3**: Comparison between group I and group III. **P4**: Comparison between group II and group III. One way ANOVA test followed by post-hoc test/LSD were or Kruskal Wallis test followed by Mann-Whitney test were used to compare between different groups

### Levels of metabolites

There was no significant difference in mean plasma urea levels among the three groups. However, there was a significant difference decrease in mean ammonia levels in patients compared to controls (Table 7).

**Table 7:** Levels of metabolites

	Group I (*n* = 26)	Group II (*n* = 22)	Group III (*n* = 38)	*P*- value^1^	*P*- value^2^	*P*- value^3^	*P*- value^4^
**Urea:**							
Mean ± SD	428.49 ± 189.98	373.78 ± 81.55	342.49 ± 90.03	0.223	0.450	0.109	0.394
Median (Range)	465.5 (92.5-699.2)	402.6 (254.1-435.8)	343.5 (179.6-551.8)				
**Ammonia**:							
Mean ± SD	92.15 ± 28.17	118.94 ± 28.64	221.99 ± 181.96	0.108	0.210	0.046*	0.465
Median (Range)	88.2 (54.5-131.8)	135.1 (85.9-135.9)	147.6 (73.3–630.0)				

**P1**: Comparison between all groups. **P2**: Comparison between group I and group II. **P3**: Comparison between group I and group III. **P4**: Comparison between group II and group III. Kruskal Wallis test followed by Mann-Whitney test were used to compare between different groups

### Correlation analyses

A significant positive correlation was observed between BRCA1 and Valine levels. In contrast, a significant negative correlation was observed between BRCA2 and Lysine plasma levels (Table 8). Also, a significant positive correlation was detected between the levels of Phosphoserine, Serine, Glycine, Phenylalanine and Ornithine and the hormone receptor status, where the most aggressive non luminal cases showed the highest level of these amino acids (Table 8).

**Table 8:** Correlation analyses of BRCA1 and 2 levels with amino acid levels and clinical data of BC patients

	BRCA 1 Conc		BRCA 2 Conc	
	*r*-value	*P*-value	*r*-value	*P*-value
**Age**	-0.035	0.864	-0.149	0.469
**Weight**	-0.308	0.126	-0.226	0.268
**Height**	-0.150	0.465	-0.217	0.286
**BMI**	-0.329	0.101	-0.098	0.633
**Urea**	-0.321	0.482	-0.500	0.253
**Ammonia**	-0.214	0.645	-0.071	0.879
**Phosphoserine**	0.286	0.535	-0.286	0.535
**Tau**	-0.571	0.180	-0.036	0.939
**Asp**	0.500	0.667	0.500	0.667
**Thr**	-0.464	0.294	0.179	0.702
**Ser**	0.143	0.760	-0.071	0.879
**Asn**	-0.179	0.702	-0.536	0.215
**Gly**	0.393	0.383	-0.643	0.119
**Ala**	0.250	0.589	-0.464	0.294
**Val**	**0.857**	0.014*	-0.607	0.148
**Met**	0.071	0.879	-0.714	0.071
**Ile**	0.179	0.702	-0.464	0.294
**Leu**	0.143	0.760	-0.536	0.215
**Tyr**	0.143	0.760	-0.143	0.760
**Phe**	0.214	0.645	-0.214	0.645
**His**	-0.036	0.939	-0.107	0.819
**Orn**	0.214	0.645	0.071	0.879
**Lys**	0.464	0.294	**-0.786**	0.036*
**Arg**	-0.600	0.208	-0.257	0.623
**Cys**	-0.371	0.468	0.714	0.111
**Tryp**	-0.657	0.156	0.600	0.208
**Glu**	0.321	0.482	-0.500	0.253
**TNM Stage**	0.272	0.178	-0.211	0.301
**Grade**	0.105	0.611	-0.291	0.149
**Family history**	0.059	0.776	-0.007	0.975
**Hormone Receptor status (according to their severity**: **non-luminal > luminal B > luminal A)**	0.191	0.351	-0.012	0.953
**Age of presentation**	-0.200	0.328	0.043	0.834

Spearman correlation was done to measure correlation between quantitative variables. Significant correlations are represented in bold font.

**Table 9:** Correlation analyses of amino acid levels and clinical data of BC patients

	TNM Stage		Family history		Hormone Receptor status		Age of presentation	
	*r*	*P* value	*r*	*P* value	*R*	*P* value	*r*	*P* value
**Urea**	-0.612	0.144	0.408	0.363	0.424	0.343	-0.607	0.148
**Ammonia**	-0.612	0.144	-0.204	0.661	0.617	0.140	0.071	0.879
**Phosphoserine**	-0.408	0.363	0.612	0.144	**0.926**	0.003*	-0.429	0.337
**Tau**	-0.612	0.144	0	1	-0.501	0.252	-0.429	0.337
**Asp**	0.866	0.333	.	.	-0.866	0.333	-0.500	0.667
**Thr**	-0.612	0.144	-0.204	0.661	0.386	0.393	0.321	0.482
**Ser**	-0.204	0.661	0.204	0.661	**0.849**	0.016*	0.071	0.879
**Asn**	-0.612	0.144	0.204	0.661	0.540	0.211	-0.107	0.819
**Gly**	-0.204	0.661	0.408	0.363	**0.810**	0.027*	-0.071	0.879
**Ala**	0.000	1	-0.204	0.661	-0.077	0.869	0.071	0.879
**Val**	0.204	0.661	0.612	0.144	0.579	0.174	-0.071	0.879
**Met**	-0.408	0.363	0.408	0.363	0.231	0.618	-0.071	0.879
**Ile**	-0.612	0.144	0.408	0.363	0.617	0.140	-0.214	0.645
**Leu**	-0.612	0.144	0.612	0.144	0.424	0.343	-0.393	0.383
**Tyr**	-0.612	0.144	0.612	0.144	0.463	0.296	-0.214	0.645
**Phe**	-0.612	0.144	0.612	0.144	**0.849**	0.016*	-0.321	0.482
**His**	-0.612	0.144	0.408	0.363	0.694	0.083	0.036	0.939
**Orn**	-0.408	0.363	0.204	0.661	**0.926**	0.003*	-0.071	0.879
**Lys**	-0.204	0.661	0.408	0.363	0.579	0.174	-0.143	0.760
**Arg**	-0.655	0.158	-0.393	0.441	0.098	0.854	0.200	0.704
**Cys**	-0.393	0.441	-0.131	0.805	-0.154	0.770	0.429	0.397
**Tryp**	-0.131	0.805	-0.655	0.158	-0.802	0.055	0.029	0.957
**Glu**	0.204	0.661	0.000	1	-0.116	0.805	-0.393	0.383

Spearman correlation was done to measure correlation between quantitative variables. Significant correlations are represented in bold font

## Discussion

Breast cancer (BC) remains a global public health problem and a leading cause of cancer mortality among women [20]. Early diagnosis of BC is limited to the classic screening tools, which have several disadvantages as high radiation risk and high cost, therefore it is necessary to search for novel reliable biomarkers that can be used for its early detection and monitoring of disease progression [21].

An increased interest in the field of metabolomics has been noticed in the last decade due to their potential for clinical applications [22–24]. Among the vast types of metabolites, plasma free amino acids (PFAA) present as promising potential disease biomarkers i for different cancers [22–25].

Under normal conditions, amino acids maintain a state of protein balance, despite continual cycling between protein synthesis and degradation [26]. However, the case differs in cancer cells where PFAAs are in high demand for protein, DNA synthesis and building new blood vessels which may lead to a lower availability of PFAAs in cancer patients [17, 27]. On the other hand, studies reported that the increase in proliferation rate in tumors maybe correlated with an increase in levels of some amino acids [17]. This heterogeneity might be due to the differences in subjects’ demographics, in addition to the different disease stages [18, 19].

The results of the present study demonstrated a significant increase in mean plasma levels of glutamate in patients compared to levels in the control group. We also noted that glutamate was significantly upregulated in patients compared to females with family history of BC. This could possibly be attributed to the fact that tumor cells consume glutamine largely aiming to produce energy through the tricarboxylic acid cycle and to maintain protein synthesis necessary for tumor growth and proliferation [17, 26, 28]. The high rate of glutamine uptake may be also due to the stimulated uptake of essential AA required to maintain the mitochondrial integrity [29]. Furthermore, findings from previous studies demonstrated an impaired ability of the peripheral skeletal muscle to extract glutamate from circulation in patients with gastrointestinal, renal, bronchial and breast cancer leading to accumulating plasma glutamate concentrations [26, 30].

Contradictory findings are reported regarding plasma alanine levels in cancer patients [14, 26, 27]. The increased levels of alanine observed in plasma of cancer patients in our study may be due to its increased production by tumor cells [17, 30]. However, these results are contradictory with those observed by Eniu et al. who showed a downregulation in alanine availability in BC patients which was attributed to its conversion to glucose in the liver [19].

Cysteine is a key contributor to metabolic remodeling associated with cancer [31]. Our results showed increased plasma cysteine levels in BC patients, which comes in line with previous studies [32]. Interestingly, plasma levels of alanine, phenylalanine, glutamate and cysteine were also increased in females with family of BC compared to controls, this could offer these AA profile as important potential early diagnostic tool of BC in Egyptian women. Further follow up is required to confirm these results.

On the other hand, there was a significant decrease in mean plasma levels of taurine, threonine, serine, glycine, valine, methionine and histidine where their plasma levels were lower in patients and relatives compared to controls. Glycine and serine are both classic glycolysis metabolites [24]. It was reported that glycine uptake and metabolism were necessary to promote tumorigenesis and malignancy [33, 34]. Additionally, serine is used for one-carbon metabolism and nucleotide synthesis which is necessary for cancer cell proliferation [35, 36] leading to a decrease in their plasma levels which is in line with our results. On the contrary, several studies reported an increase in serine levels in patients with pancreatic cancer, BC and colorectal cancer [14, 17, 26, 37] which was explained by the increased enzymatic activity involved in serine biosynthesis in tumor cells [38].

Our results showed a significant decrease in valine levels in BC patients compared to control females, however we detected no significant differences in leucine and isoleucine levels. Branched chain AAs (BCAAs) are major contributors in the maintenance of lean body mass, and they play a major role in stimulation of skeletal muscle protein synthesis through activation of mTOR [39, 40]. Lower plasma valine concentrations in BC patients may be due to increased uptake by peripheral muscle tissue. Indeed these results are in agreement with previous studies [41, 42], however, others reported high plasma valine concentrations observed in cancer patients which could be attributed to the enhanced breakdown of body protein shown in those cancer patients [39, 43].

In the present study, we show low levels of taurine in BC patients. Taurine was suggested previously as an early diagnosis biomarker for malignant changes in the breast [44]. We also show low levels of histidine and threonine in BC patients in contradiction with Barnes et al., who reported less histidine uptake in breast cancer patients [26]. Histidine is used to synthesize carnosine, an antioxidant that suppresses cancer proliferation [45]. The increased uptake of histidine in our patients’ tissues may be due to increased tumor induced-oxidative damage in the tissue. As for the lower cancer threonine levels, it was reported that it might be due to increased production of pyruvate from threonine associated with the impaired glutamate uptake as seen in our patients [46].

BRCA1 and BRCA2 genes stand out among DNA repair pathway genes for their specific relationship with BC [47, 48]. Upregulated expression of BRCA1 and BRCA2 genes was reported in BC and high BRCA1 gene expression was demonstrated to increase the risk of early distant metastasis in ER + breast cancer patients [8, 9]. Moreover, a worse overall survival rate was substantially correlated with overexpression of BRCA1/2 [49].

Our study results showed significant elevations in BRCA1 and 2 protein levels in sera of patients and of females with family history compared to healthy females with a significant upregulation of BRCA2 levels in patients compared to females with family history. Also, a significant positive correlation was observed between plasma BRCA1 and Valine levels. In contrast, a significant negative correlation was observed between BRCA2 and Lysine plasma levels. To the best of our knowledge, this is the first study to explore the correlation between BRCA 1&2 and amino acids level in BC.

In conclusion, this study results shows that PFAAs profile can potentially help in early screening for BC patients and for susceptible females. Furthermore, combination of BRCA 1 and 2 assessment and AAs profiling could have a strong diagnostic potential for BC. Further studies are needed to validate this assumption.

### Limitations

One of the limitations of this study is the sole assessment of BRCA1 and BRCA2 serum protein levels and not at the gene level. Further research that measures both mRNA and protein levels is essential for a comprehensive understanding of the molecular pathogenesis of breast cancer. Other limitations include lack of follow up in addition to the small sample size. Further studies with larger sample size and follow up are needed to check if the levels of PFAA are associated with risk of development of BC in family subjects and to fully understand the effect of changes in PFAAs particularly in connection to BRCA1,2 genes expression which could provide further insight on their role as prognostic and diagnostic biomarkers of BC.

## Data Availability

All related data and materials are available from the corresponding author upon request.

## References

[1] Sung H, Ferlay J, Siegel RL, Laversanne M, Soerjomataram I, Jemal A et al (2021) Global Cancer statistics 2020: GLOBOCAN estimates of incidence and Mortality Worldwide for 36 cancers in 185 countries. CA Cancer J Clin 71(3):209–249. 10.3322/caac.21660 33538338 10.3322/caac.21660

[2] Siegel RL, Miller KD, Fuchs HE, Jemal A, Cancer statistics (2022) CA Cancer J Clin. 2022;72(1):7–33. 10.3322/caac.21708 10.3322/caac.21708 35020204

[3] Lozano R, Fullman N, Mumford JE, Knight M, Barthelemy CM, Abbafati C, et al. Measuring universal health coverage based on an index of effective coverage of health services in 204 countries and territories, 1990–2019: a systematic analysis for the Global Burden of Disease Study 2019. The Lancet. 2020;396(10258):1250-84. 10.1016/S0140-6736(20)30750-9 10.1016/S0140-6736(20)30750-9 PMC7562819 32861314

[4] Ibrahim AS, Khaled HM, Mikhail NN, Baraka H, Kamel H (2014) Cancer incidence in Egypt: results of the national population-based cancer registry program. J Cancer Epidemiol 2014:437971. 10.1155/2014/437971 25328522 10.1155/2014/437971 PMC4189936

[5] Łukasiewicz S, Czeczelewski M, Forma A, Baj J, Sitarz R, Stanisławek A, Breast, Cancer—Epidemiology (2021) Risk factors, classification, prognostic markers, and current treatment Strategies—An. Updated Rev Cancers 13(17):4287 10.3390/cancers13174287 PMC8428369 34503097

[6] Abu-Helalah M, Azab B, Mubaidin R, Ali D, Jafar H, Alshraideh H et al (2020) BRCA1 and BRCA2 genes mutations among high risk Breast cancer patients in Jordan. Sci Rep 10(1):17573. 10.1038/s41598-020-74250-2 33067490 10.1038/s41598-020-74250-2 PMC7568559

[7] Hosey AM, Gorski JJ, Murray MM, Quinn JE, Chung WY, Stewart GE et al (2007) Molecular basis for Estrogen Receptor α Deficiency in BRCA1-Linked Breast Cancer. JNCI: J Natl Cancer Inst 99(22):1683–1694. 10.1093/jnci/djm207 18000219 10.1093/jnci/djm207 PMC6485437

[8] Roy R, Chun J, Powell SN (2011) BRCA1 and BRCA2: different roles in a common pathway of genome protection. Nat Rev Cancer 12(1):68–78. 10.1038/nrc3181 22193408 10.1038/nrc3181 PMC4972490

[9] Chang H-J, Yang U-C, Lai M-Y, Chen C-H, Fann Y-C (2022) High BRCA1 gene expression increases the risk of early distant Metastasis in ER + breast cancers. Sci Rep 12(1):77. 10.1038/s41598-021-03471-w 34996912 10.1038/s41598-021-03471-w PMC8741892

[10] Farouk O, Ebrahim MA, Senbel A, Emarah Z, Abozeed W, Seisa MO et al (2016) Breast cancer characteristics in very young Egyptian women ≤ 35 years. Breast Cancer (Dove Med Press) 8:53–58. 10.2147/bctt.S99350 27103842 10.2147/BCTT.S99350 PMC4827892

[11] El Saghir NS, Khalil MK, Eid T, El Kinge AR, Charafeddine M, Geara F et al (2007) Trends in epidemiology and management of Breast cancer in developing arab countries: a literature and registry analysis. Int J Surg 5(4):225–233. 10.1016/j.ijsu.2006.06.015 17660128 10.1016/j.ijsu.2006.06.015

[12] Abdelaziz AH, Shawki MA, Shaaban AM, Albarouki SK, Rachid AM, Alsalhani OM et al (2021) Breast Cancer awareness among Egyptian women and the impact of caring for patients with Breast Cancer on Family caregivers’ knowledge and Behaviour. Res Oncol 17(1):1–8. 10.21608/resoncol.2020.42340.1114

[13] Abdelaziz AH, Abdou AM, Habeeb CN (2018) 1571P - Breast cancer treatment waiting time, patient and provider contributions: an Egyptian Breast cancer centre experience. Ann Oncol 29:viii566. 10.1093/annonc/mdy297.015

[14] Miyagi Y, Higashiyama M, Gochi A, Akaike M, Ishikawa T, Miura T et al (2011) Plasma free amino acid profiling of five types of cancer patients and its application for early detection. PLoS ONE 6(9):e24143. 10.1371/journal.pone.0024143 21915291 10.1371/journal.pone.0024143 PMC3168486

[15] Saoi M, Britz-McKibbin P (2021) New advances in tissue metabolomics: a review. Metabolites 11(10). 10.3390/metabo11100672 10.3390/metabo11100672 PMC8541552 34677387

[16] Kim YH, Shim HS, Kim KH, Lee J, Chung BC, Kowall NW et al (2019) Metabolomic analysis identifies alterations of amino acid Metabolome signatures in the Postmortem Brain of Alzheimer’s Disease. Exp Neurobiol 28(3):376–389. 10.5607/en.2019.28.3.376 31308797 10.5607/en.2019.28.3.376 PMC6614073

[17] Poschke I, Mao Y, Kiessling R, de Boniface J (2013) Tumor-dependent increase of serum amino acid levels in Breast cancer patients has diagnostic potential and correlates with molecular Tumor subtypes. J Transl Med 11:290. 10.1186/1479-5876-11-290 24237611 10.1186/1479-5876-11-290 PMC3835137

[18] Bi X, Henry CJ (2017) Plasma-free amino acid profiles are predictors of cancer and Diabetes development. Nutr Diabetes 7(3):e249. 10.1038/nutd.2016.55 28287627 10.1038/nutd.2016.55 PMC5380892

[19] Eniu DT, Romanciuc F, Moraru C, Goidescu I, Eniu D, Staicu A et al (2019) The decrease of some serum free amino acids can predict Breast cancer diagnosis and progression. Scand J Clin Lab Invest 79(1–2):17–24. 10.1080/00365513.2018.1542541 30880483 10.1080/00365513.2018.1542541

[20] Siegel RL, Miller KD, Jemal A, Cancer statistics (2020) CA: A Cancer Journal for Clinicians. 2020;70(1):7–30. 10.3322/caac.21590 10.3322/caac.21590 31912902

[21] Wang L (2017) Early diagnosis of Breast Cancer. Sensors 17(7):1572 28678153 10.3390/s17071572 PMC5539491

[22] Pietkiewicz D, Klupczynska-Gabryszak A, Plewa S, Misiura M, Horala A, Miltyk W et al (2021) Free amino acid alterations in patients with gynecological and Breast Cancer: a review. Pharmaceuticals 14(8):731 34451829 10.3390/ph14080731 PMC8400482

[23] Liu D-H, Wen G-M, Song C-L, Ji L-J, Xia P (2022) Amino acid profiles in the tissue and serum of patients with Liver cancer. Open Med 17(1):1797–1802. 10.1515/med-2022-0589 10.1515/med-2022-0589 PMC9675036 36447523

[24] Bi X, Henry CJ (2017) Plasma-free amino acid profiles are predictors of cancer and Diabetes development. Nutr Diabetes 7(3):e249–e. 10.1038/nutd.2016.55 28287627 10.1038/nutd.2016.55 PMC5380892

[25] Noguchi Y, Zhang QW, Sugimoto T, Furuhata Y, Sakai R, Mori M et al (2006) Network analysis of plasma and tissue amino acids and the generation of an amino index for potential diagnostic use. Am J Clin Nutr 83(2):513s–9s. 10.1093/ajcn/83.2.513S 16470023 10.1093/ajcn/83.2.513S

[26] Barnes T, Bell K, DiSebastiano KM, Vance V, Hanning R, Russell C et al (2014) Plasma amino acid profiles of Breast cancer patients early in the trajectory of the Disease differ from healthy comparison groups. Appl Physiol Nutr Metab 39(6):740–744. 10.1139/apnm-2013-0526 24819038 10.1139/apnm-2013-0526

[27] Proenza AM, Oliver J, Palou A, Roca P (2003) Breast and Lung cancer are associated with a decrease in blood cell amino acid content. J Nutr Biochem 14(3):133–138. 10.1016/s0955-2863(02)00225-5 12742540 10.1016/s0955-2863(02)00225-5

[28] Nagata C, Wada K, Tsuji M, Hayashi M, Takeda N, Yasuda K (2014) Plasma amino acid profiles are associated with biomarkers of Breast cancer risk in premenopausal Japanese women. Cancer Causes Control 25(2):143–149. 10.1007/s10552-013-0316-8 24186145 10.1007/s10552-013-0316-8

[29] Alberghina L, Gaglio D (2014) Redox control of glutamine utilization in cancer. Cell Death Dis 5(12):e1561. 10.1038/cddis.2014.513 25476909 10.1038/cddis.2014.513 PMC4454159

[30] Mourtzakis M, Graham TE, González-Alonso J, Saltin B (2008) Glutamate availability is important in intramuscular amino acid metabolism and TCA cycle intermediates but does not affect peak oxidative metabolism. J Appl Physiol (1985) 105(2):547–554. 10.1152/japplphysiol.90394.2008 18511521 10.1152/japplphysiol.90394.2008

[31] Bonifácio VDB, Pereira SA, Serpa J, Vicente JB (2021) Cysteine metabolic circuitries: druggable targets in cancer. Br J Cancer 124(5):862–879. 10.1038/s41416-020-01156-1 33223534 10.1038/s41416-020-01156-1 PMC7921671

[32] Lin J, Lee I-M, Song Y, Cook NR, Selhub J, Manson JE et al (2010) Plasma homocysteine and cysteine and risk of Breast Cancer in women. Cancer Res 70(6):2397–2405. 10.1158/0008-5472.Can-09-3648 20197471 10.1158/0008-5472.CAN-09-3648 PMC2840179

[33] Jain M, Nilsson R, Sharma S, Madhusudhan N, Kitami T, Souza AL et al (2012) Metabolite profiling identifies a key role for glycine in rapid cancer cell proliferation. Science 336(6084):1040–1044. 10.1126/science.1218595 22628656 10.1126/science.1218595 PMC3526189

[34] Leichtle AB, Nuoffer JM, Ceglarek U, Kase J, Conrad T, Witzigmann H et al (2012) Serum amino acid profiles and their alterations in Colorectal cancer. Metabolomics 8(4):643–653. 10.1007/s11306-011-0357-5 22833708 10.1007/s11306-011-0357-5 PMC3397217

[35] Amelio I, Cutruzzolá F, Antonov A, Agostini M, Melino G (2014) Serine and glycine metabolism in cancer. Trends Biochem Sci 39(4):191–198. 10.1016/j.tibs.2014.02.004 24657017 10.1016/j.tibs.2014.02.004 PMC3989988

[36] Possemato R, Marks KM, Shaul YD, Pacold ME, Kim D, Birsoy K et al (2011) Functional genomics reveal that the serine synthesis pathway is essential in Breast cancer. Nature 476(7360):346–350. 10.1038/nature10350 21760589 10.1038/nature10350 PMC3353325

[37] Fukutake N, Ueno M, Hiraoka N, Shimada K, Shiraishi K, Saruki N et al (2015) A novel Multivariate Index for Pancreatic Cancer Detection based on the plasma free amino Acid Profile. PLoS ONE 10(7):e0132223. 10.1371/journal.pone.0132223 26133769 10.1371/journal.pone.0132223 PMC4489861

[38] Medina MA, Márquez J, Núñez de Castro I (1992) Interchange of amino acids between Tumor and host. Biochem Med Metab Biol 48(1):1–7. 10.1016/0885-4505(92)90041-v 1524866 10.1016/0885-4505(92)90041-v

[39] Mayers JR, Wu C, Clish CB, Kraft P, Torrence ME, Fiske BP et al (2014) Elevation of circulating branched-chain amino acids is an early event in human pancreatic adenocarcinoma development. Nat Med 20(10):1193–1198. 10.1038/nm.3686 25261994 10.1038/nm.3686 PMC4191991

[40] Kimball SR, Jefferson LS (2006) Signaling pathways and molecular mechanisms through which branched-chain amino acids mediate translational control of protein synthesis. J Nutr 136(1 Suppl):227s–31s. 10.1093/jn/136.1.227S 16365087 10.1093/jn/136.1.227S

[41] Qiu Y, Cai G, Su M, Chen T, Zheng X, Xu Y et al (2009) Serum metabolite profiling of human Colorectal cancer using GC-TOFMS and UPLC-QTOFMS. J Proteome Res 8(10):4844–4850. 10.1021/pr9004162 19678709 10.1021/pr9004162

[42] Budhathoki S, Iwasaki M, Yamaji T, Yamamoto H, Kato Y, Tsugane S (2017) Association of plasma concentrations of branched-chain amino acids with risk of colorectal adenoma in a large Japanese population. Ann Oncol 28(4):818–823. 10.1093/annonc/mdw680 28011449 10.1093/annonc/mdw680

[43] Connell TM (2013) The Complex role of branched chain amino acids in Diabetes and Cancer. Metabolites. 10.3390/metabo3040931 10.3390/metabo3040931 PMC3937834 24958258

[44] El Agouza IM, Eissa SS, El Houseini MM, El-Nashar DE, Abd El Hameed OM (2011) Taurine: a novel Tumor marker for enhanced detection of Breast cancer among female patients. Angiogenesis 14(3):321–330. 10.1007/s10456-011-9215-3 21553281 10.1007/s10456-011-9215-3

[45] Gaunitz F, Hipkiss AR (2012) Carnosine and cancer: a perspective. Amino Acids 43(1):135–142. 10.1007/s00726-012-1271-5 22454085 10.1007/s00726-012-1271-5

[46] Engelen MP, Schols AM, Does JD, Deutz NE, Wouters EF (2000) Altered glutamate metabolism is associated with reduced muscle glutathione levels in patients with Emphysema. Am J Respir Crit Care Med 161(1):98–103. 10.1164/ajrccm.161.1.9901031 10619804 10.1164/ajrccm.161.1.9901031

[47] Murthy P, Muggia F (2019) Women’s cancers: how the discovery of BRCA genes is driving current concepts of cancer biology and therapeutics. Ecancermedicalscience 13:904. 10.3332/ecancer.2019.904 30915162 10.3332/ecancer.2019.904 PMC6411414

[48] Mehrgou A, Akouchekian M (2016) The importance of BRCA1 and BRCA2 genes mutations in Breast cancer development. Med J Islam Repub Iran 30:369 27493913 PMC4972064

[49] Musolino A, Naldi N, Michiara M, Bella MA, Zanelli P, Bortesi B et al (2005) A Breast cancer patient from Italy with germline mutations in both the BRCA1 and BRCA2 genes. Breast Cancer Res Treat 91(2):203–205. 10.1007/s10549-004-7704-4 15868448 10.1007/s10549-004-7704-4

